# Supplementary material for: Investigation of UTR Variants by Computational Approaches Reveal Their Functional Significance in PRKCI Gene Regulation
Source: Genes (Basel). 2023 Jan 18;14(2):247. doi: 10.3390/genes14020247 (PMC9956319; doi:10.3390/genes14020247)
Supplement: Supplementary file 1 [file genes-14-00247-s001.zip › genes-2136973-supplementary.pdf]

Table S1. List of 3 and 5 prime UTRs from Ensembl.

Variant ID	vf	Location	Chr: bp	vf_allele
rs376442140	1.03E+08	3:170222425	3:170222425	C
rs550470925	1.05E+08	3:170222426	3:170222426	A
rs1458097555	6.58E+08	3:170222429	3:170222429-170222434	AGG
rs568661404	1.12E+08	3:170222433	3:170222433	A
rs1259735324	5.27E+08	3:170222434	3:170222434	A
rs1207991709	3.77E+08	3:170222437	3:170222437	C
rs1560162557	6.76E+08	3:170222438	3:170222438	T
rs902629612	1.33E+08	3:170222440	3:170222440	A
rs773097863	1.22E+08	3:170222441	3:170222441	A
rs1273608579	4.91E+08	3:170222444	3:170222444	A
rs1056686732	7.31E+08	3:170222445	3:170222445	G
rs113857684	94012889	3:170222446	3:170222446	G
rs904649847	1.34E+08	3:170222447	3:170222447	C
rs1446312225	6.57E+08	3:170222448	3:170222448	A
rs1378180396	5.77E+08	3:170222449	3:170222449	T
rs1300233715	5.17E+08	3:170222450	3:170222450	A
rs1446501543	6.57E+08	3:170222453	3:170222453	G
rs1359048202	5.62E+08	3:170222455	3:170222455	A
rs1176177940	4.24E+08	3:170222457	3:170222457	G
rs1012510381	2.48E+08	3:170222463	3:170222463	A
rs1415621271	6.1E+08	3:170222465	3:170222465	T
rs1024270582	2.6E+08	3:170222468	3:170222468	T
rs99943859	2.04E+0	3:1702224	3:170222469	A

6	8	69		
rs10316896	2.64E+0	3:1702224		
97	8	70	3:170222470	T
rs37091169		3:1702224		
0	1E+08	71	3:170222471	G
rs15773335	6.82E+0	3:1702224		
42	8	73	3:170222473	G
rs99488464	2.02E+0	3:1702224		
2	8	82	3:170222482	C
rs14828986	6.67E+0	3:1702224		
17	8	90	3:170222490	T
rs12379259	4.23E+0	3:1702224		
54	8	91	3:170222491	T
rs15773335	6.82E+0	3:1702224		
58	8	92	3:170222492	C
rs14574283	6.58E+0	3:1702224		
32	8	93	3:170222493	G
rs10272627	2.61E+0	3:1702224		
10	8	94	3:170222494	C
rs11178204	9306697	3:1702224		
1	7	95	3:170222495	A
rs98315979	7.16E+0	3:1702225		
1	8	01	3:170222501	G
rs12397488	4.59E+0	3:1702225		
69	8	02	3:170222502	A
rs12549139	5.24E+0	3:1702225		
40	8	04	3:170222504	T
rs13096385	5.22E+0	3:1702225		
67	8	06	3:170222506	C
rs12269098	4.17E+0	3:1702225		
82	8	09	3:170222509	G
rs86761368	1.24E+0	3:1702225		
2	8	10	3:170222510	A
rs96511027	1.56E+0	3:1702225		
3	8	11	3:170222511	A
rs11725472	4.22E+0	3:1702225		
10	8	12	3:170222512	T
rs14208429	6.14E+0	3:1702225		
39	8	13	3:170222513	A
rs14120539	6.06E+0	3:1702225		
50	8	14	3:170222514	A
rs97535571	1.77E+0	3:1702225		
5	8	15	3:170222515	G
rs96623644	1.71E+0	3:1702225		
2	8	18	3:170222518	A
rs86631279	1.24E+0	3:1702225		
2	8	19	3:170222519	T
rs13817249	5.79E+0	3:1702225	3:170222519	-

86	8	19		
rs95254676	1.49E+0	3:1702225		
1	8	20	3:170222520	T
rs14142465		3:1702225		
35	6.1E+08	22	3:170222522	T
rs11835800	3.53E+0	3:1702225		
02	8	23	3:170222523	T
rs92181936		3:1702225		
8	1.4E+08	24	3:170222524	C
rs12611657	5.29E+0	3:1702225		
75	8	30	3:170222530	G
rs12159227		3:1702225		
11	3.9E+08	34	3:170222534	C
rs98303745	7.15E+0	3:1702225		
5	8	40	3:170222540	A
rs94664238	1.48E+0	3:1702225		
1	8	41	3:170222541	C
rs54823286	1.04E+0	3:1702225		
6	8	47	3:170222547	C
rs13215583	5.35E+0	3:1702225		
27	8	48	3:170222548	A
rs13138525	5.24E+0	3:1702225		
83	8	51	3:170222551	T
rs93839863	1.44E+0	3:1702225		
2	8	53	3:170222553	A
rs13390340	5.49E+0	3:1702225		
75	8	54	3:170222554	T
rs56647765	1.11E+0	3:1702225		
8	8	58	3:170222558	C
rs14411373	6.52E+0	3:1702225		
71	8	61	3:170222561	T
rs12770404	4.93E+0	3:1702225		
59	8	62	3:170222562	C
rs14000172	5.95E+0	3:1702225		
92	8	63	3:170222563-170222569	CGGACGGACGG
rs13866898	5.82E+0	3:1702225		
24	8	66	3:170222566	G
rs12914965	5.16E+0	3:1702225		
66	8	67	3:170222567	A
rs53376248	1.03E+0	3:1702225		
8	8	71	3:170222571	A
rs93543739	1.46E+0	3:1702225		
3	8	72	3:170222572	C
rs15773336	6.82E+0	3:1702225		
57	8	73	3:170222573	A
rs91557676	1.36E+0	3:1702225		
1	8	74	3:170222574	A
rs11930512	3.58E+0	3:1702225	3:170222575	A

32	8	75		
rs10565303	7.31E+0	3:1702225		
65	8	78	3:170222578	T
rs14782294	6.67E+0	3:1702225		
73	8	79	3:170222579	A
rs89665728		3:1702225		
3	1.3E+08	80	3:170222580	G
rs55831833	1.07E+0	3:1702225		
3	8	81	3:170222581	G
rs10128071	2.48E+0	3:1702225		
49	8	82	3:170222582	A
rs90478631	1.34E+0	3:1702225		
0	8	86	3:170222586	G
rs14945985	9767052	3:1702225		
3	9	87	3:170222587	A
rs86767638	1.24E+0	3:1702225		
6	8	88	3:170222588	A
rs89065491	1.28E+0	3:1702225		
2	8	93	3:170222593	A
rs12571454	5.26E+0	3:1702225		
35	8	94	3:170222594	G
rs10089310		3:1702226		
62	2.1E+08	06	3:170222606	T
rs12146047	3.89E+0	3:1702226		
50	8	07	3:170222607	G
rs12579028	5.26E+0	3:1702226		
05	8	11	3:170222611	T
rs99569518	2.02E+0	3:1702226		
4	8	13	3:170222613	T
rs12985210	5.19E+0	3:1702226		
72	8	14	3:170222614	T
rs10273135	2.61E+0	3:1702226		
57	8	16	3:170222616	G
rs95037803		3:1702226		
8	1.5E+08	17	3:170222617	T
rs12993726	5.16E+0	3:1702226		
77	8	21	3:170222621	T
rs77286167	1.22E+0	3:1702226		
6	8	21	3:170222621-170222631	CGC
rs15601626	6.76E+0	3:1702226		
90	8	22	3:170222622	T
rs19958450	9780902	3:1702226		
3	5	23	3:170222623	T
rs76250298	1.21E+0	3:1702226		
6	8	23	3:170222623-170222629	CCCCCC
rs15538324	6.73E+0	3:1702226		
19	8	23	3:170222623-170222637	CCCCCCCGCACCCCCCCGCACCCCC
rs14349104	6.48E+0	3:1702226	3:170222624	G

46		06		
rs89098335	1.29E+0	3:1703035		
5	8	09	3:170303509	G
rs13938866	5.92E+0	3:1703035		
08	8	10	3:170303510	C
rs14010789	5.96E+0	3:1703035		
99	8	11	3:170303511	T
rs10066265	2.09E+0	3:1703035		
81	8	32	3:170303532	G
rs14291069	6.17E+0	3:1703035		
22	8	41	3:170303541	C
rs12699592	4.89E+0	3:1703035		
56	8	42	3:170303542	C
rs15773809	6.82E+0	3:1703035		
58	8	45	3:170303545	C
rs11579653	7.32E+0	3:1703035		
99	8	52	3:170303552	C
rs14402581	6.52E+0	3:1703035		
99	8	53	3:170303553	A
rs13818655	5.79E+0	3:1703035		
09	8	55	3:170303555	G
rs37528330	1.02E+0	3:1703035		
0	8	57	3:170303557	A
rs14408615	6.52E+0	3:1703035		
79	8	68	3:170303568	G
rs12557095	5.25E+0	3:1703035		
67	8	73	3:170303573	G
rs96239670	1.54E+0	3:1703035		
3	8	74	3:170303574	C
rs14842683	6.68E+0	3:1703035		
78	8	77	3:170303577	C
rs12347471	4.21E+0	3:1703035		
48	8	83	3:170303583	C
rs12839906	4.96E+0	3:1703035		
02	8	86	3:170303586	G
rs56458673		3:1703035		
2	1.1E+08	87	3:170303587	T
rs15773810	6.82E+0	3:1703035		
00	8	88	3:170303588-170303593	TT
rs12909893	5.15E+0	3:1703035		
49	8	95	3:170303595	C
rs97418480	1.76E+0	3:1703036		
4	8	06	3:170303606	A
	9236632	3:1703036		
rs3903277	1	08	3:170303608	G
rs13550800		3:1703036		
64	5.6E+08	13	3:170303613	C
rs13118487	5.23E+0	3:1703036	3:170303619	C

Table S2. Regulome DB score for 3 prime UTRs.

Chromosome location	dbSNP IDs	Rank	Score
chr3:170020920..170020921	rs1380292764	5	0.13454
chr3:170020928..170020929	rs760864637	5	0.13454
chr3:170020930..170020931	rs771089704	5	0.13454
chr3:170020931..170020932	rs1161314552	5	0.13454
chr3:170020932..170020933	rs767691077	5	0.13454
chr3:170020935..170020936	rs145216751	5	0.13454
chr3:170020943..170020944	rs776895087	5	0.13454
chr3:170020944..170020945	rs762966781	5	0.13454
chr3:170020954..170020955	rs1368584618	5	0.13454
chr3:170020956..170020957	rs1359988916	5	0.13454
chr3:170020958..170020959	rs766444327	5	0.13454
chr3:170020959..170020960	rs751362718	5	0.13454
chr3:170020960..170020961	rs533723433	5	0.13454
chr3:170020962..170020963	rs766502265	5	0.13454
chr3:170020964..170020965	rs1446086302	5	0.13454
chr3:170020965..170020966	rs767271191	5	0.13454
chr3:170020971..170020972	rs1379832994	5	0.13454
chr3:170020975..170020976	rs187988898	5	0.13454
chr3:170020980..170020981	rs867514822	5	0.13454
chr3:170020987..170020988	rs1022664516	5	0.13454
chr3:170020989..170020990	rs1181668376	5	0.13454
chr3:170020990..170020991	rs1483347012	5	0.13454
chr3:170020996..170020997	rs902338303	5	0.13454
chr3:170021012..170021013	rs570181892	5	0.33738
chr3:170021017..170021018	rs749748914, rs999400234	5	0.44483
chr3:170021019..170021020	rs1441775919	5	0.0354
chr3:170021028..170021029	rs1029505852	5	0.58
chr3:170021031..170021032	rs537460553	5	0.26167
chr3:170021033..170021034	rs555843406	5	0
chr3:170021035..170021036	rs955074863	5	0.13454
chr3:170021036..170021037	rs1381122178	5	0.13454
chr3:170021040..170021041	rs1440812344	5	0.13454
chr3:170021043..170021044	rs1271215689	5	0.13454
chr3:170021044..170021045	rs1364536889	5	0.13454
chr3:170021050..170021051	rs368763273	5	0.13454
chr3:170021052..170021053	rs1422012162	5	0.17392
chr3:170021057..170021058	rs1364279887	5	0.71614
chr3:170021058..170021059	rs1164757827	5	0
chr3:170021067..170021068	rs1017916630	5	0.00347
chr3:170021068..170021069	rs1424732871	5	0
chr3:170021075..170021076	rs759769254	6	0.20829

chr3:170021080..170021081	rs962715050	6	0.11318
chr3:170021081..170021082	rs765619428	6	0.16346
chr3:170021082..170021083	rs373110439	6	0.5804
chr3:170021090..170021091	rs1489666049	6	0.53625
chr3:170021092..170021093	rs1271885153	6	0.49716
chr3:170021095..170021096	rs1264762631	6	0.67385
chr3:170021103..170021104	rs536090741	7	0.18412
chr3:170021105..170021106	rs1335036352	7	0.18412
chr3:170021115..170021116	rs974338230	6	0.11093
chr3:170021119..170021120	rs1215078358	6	0.21214
chr3:170021128..170021129	rs918867925	7	0.18412
chr3:170021132..170021133	rs930156449	7	0.18412
chr3:170021140..170021141	rs1340155100	6	0.33117
chr3:170021147..170021148	rs1278864720	6	0.59153
chr3:170021148..170021149	rs1270718845	6	0.25986
chr3:170021157..170021158	rs981534335	7	0.18412
chr3:170021158..170021159	rs1468032767	7	0.18412
chr3:170021159..170021160	rs71277165	7	0.18412
chr3:170021163..170021164	rs1168991721	7	0.18412
chr3:170021165..170021166	rs1464743304	7	0.18412
chr3:170021168..170021169	rs1423866639	6	0.67385
chr3:170021191..170021192	rs1185983927, rs1195450342	6	0.40391
chr3:170021194..170021195	rs1191639426, rs199720571	6	0.15852
chr3:170021195..170021196	rs1220767732	6	0.35857
chr3:170021203..170021204	rs937395657	6	0
chr3:170021204..170021205	rs1300695881	6	0.24967
chr3:170021205..170021206	rs1424747700	6	0.20016
chr3:170021206..170021207	rs1055822853, rs1329668895, rs1553846571	6	0.47449
chr3:170021207..170021208	rs1185674453	6	0.25543
chr3:170021208..170021209	rs551843301	6	0.25543
chr3:170021209..170021210	rs1464242161	6	0.72948
chr3:170021211..170021212	rs1376506225	6	0
chr3:170021215..170021216	rs1462150077	6	0.58141
chr3:170021218..170021219	rs947411361	7	0.18412
chr3:170021223..170021224	rs1434784298	7	0.18412
chr3:170021243..170021244	rs1167271298	7	0.18412
chr3:170021244..170021245	rs190906216	7	0.18412
chr3:170021247..170021248	rs371009981	7	0.18412
chr3:170021249..170021250	rs1458045300	6	0.22339
chr3:170021256..170021257	rs183165714	6	0.18347
chr3:170021260..170021261	rs1319579493	6	0.40391

chr3:170021261..170021262	rs1482402453	6	0.34125
chr3:170021264..170021265	rs1255451371	6	0
chr3:170021271..170021272	rs999452468	6	0.49716
chr3:170021288..170021289	rs1029473149	6	0.25986
chr3:170021289..170021290	rs1307700944	6	0.25986
chr3:170021293..170021294	rs1240135146	6	0.08083
chr3:170021296..170021297	rs890983355	6	0.09881
chr3:170021297..170021298	rs1393886608	6	0.24039
chr3:170021298..170021299	rs1401078999	6	0.43133
chr3:170021319..170021320	rs1006626581	6	0.48494
chr3:170021328..170021329	rs1429106922	6	0
chr3:170021329..170021330	rs1269959256	6	0.11093
chr3:170021339..170021340	rs1157965399	6	0.8051
chr3:170021340..170021341	rs1440258199	6	0.1131
chr3:170021342..170021343	rs1381865509	6	0.23675
chr3:170021344..170021345	rs375283300	6	0.23675
chr3:170021355..170021356	rs1440861579	7	0.18412
chr3:170021360..170021361	rs1255709567	7	0.18412
chr3:170021361..170021362	rs962396703	7	0.18412
chr3:170021364..170021365	rs1484268378	7	0.18412
chr3:170021370..170021371	rs1234747148	7	0.18412
chr3:170021373..170021374	rs1283990602	7	0.18412
chr3:170021374..170021375	rs564586732	7	0.18412
chr3:170021382..170021383	rs1290989349	6	0.43272
chr3:170021393..170021394	rs974184804	7	0.18412
chr3:170021395..170021396	rs3903277	7	0.18412
chr3:170021400..170021401	rs1355080064	6	0.58141
chr3:170021406..170021407	rs1311848702	6	0.34125
chr3:170021415..170021416	rs1243039412	6	0
chr3:170021417..170021418	rs1340286984	6	0.27419
chr3:170021430..170021431	rs1205156463	6	0.67385
chr3:170021432..170021433	rs1286297115	6	0.49716
chr3:170021437..170021438	rs1445180529	6	0.00333
chr3:170021446..170021447	rs377711012, rs758616548	5	0.58955
chr3:170021449..170021450	rs115170199	5	1
chr3:170021454..170021455	rs543843298	5	0.86333
chr3:170021455..170021456	rs3896073	5	1
chr3:170021458..170021459	rs368341963	5	0.2541
chr3:170021475..170021476	rs1472340224	5	0.58955
chr3:170021480..170021481	rs1190346434	5	0.58955
chr3:170021483..170021484	rs1188438028	5	0.58955
chr3:170021485..170021486	rs1415889030	5	0.58955
chr3:170021492..170021493	rs1560189190	5	0.58955

chr3:170021499..170021500	rs1261067066	5	0.58955
chr3:170021500..170021501	rs187434851	5	0.11067
chr3:170021501..170021502	rs1488685235	5	0.611
chr3:170021511..170021512	rs981670343	5	0.36674
chr3:170021514..170021515	rs763663493	5	0.992
chr3:170021516..170021517	rs1266421828	5	0.54083
chr3:170021520..170021521	rs937541417	5	0.16883
chr3:170021524..170021525	rs1338007608	5	0.58955
chr3:170021531..170021532	rs1276966304	5	0.58955
chr3:170021535..170021536	rs1399455452	5	0.58955
chr3:170021540..170021541	rs1341879308	5	0.76571
chr3:170021543..170021544	rs1321492319	5	0.195
chr3:170021554..170021555	rs1298049213	5	0.58955
chr3:170021555..170021556	rs1376452096	5	0.58955
chr3:170021557..170021558	rs1164812986	5	0.54974
chr3:170021558..170021559	rs1463162506	5	0.55717
chr3:170021559..170021560	rs991528486	5	0.76455
chr3:170021560..170021561	rs529276554	5	0.85633
chr3:170021562..170021563	rs1310720826	5	1
chr3:170021574..170021575	rs1339472269	5	0.88556
chr3:170021586..170021587	rs1197307107	5	0.58955
chr3:170021588..170021589	rs548220416	5	0.58955
chr3:170021589..170021590	rs560373226	5	0.58955
chr3:170021590..170021591	rs527504768	5	0.58955
chr3:170021592..170021593	rs1215967669	5	0.58955
chr3:170021595..170021596	rs924649425	5	0.98162
chr3:170021598..170021599	rs1256996721	5	0.88556
chr3:170021600..170021601	rs1218924627	5	1
chr3:170021609..170021610	rs1319314361	5	0.58955
chr3:170021611..170021612	rs372270690	5	0.58955
chr3:170021614..170021615	rs1051032595	5	0.58955
chr3:170021619..170021620	rs1331629155	5	0.5421
chr3:170021621..170021622	rs1280884653	5	1
chr3:170021626..170021627	rs1444266440	5	0.94308
chr3:170021631..170021632	rs890949418	5	0.58955
chr3:170021640..170021641	rs1006762786	5	0.58955
chr3:170021643..170021644	rs776277717	5	0.58955
chr3:170021644..170021645	rs1039451755	5	0.58955
chr3:170021645..170021646	rs143395024	5	0.58955
chr3:170021646..170021647	rs1477960916	5	0.58955
chr3:170021647..170021648	rs1378696658	5	0.58955
chr3:170021651..170021652	rs1196101927	5	0.58955
chr3:170021652..170021653	rs146869487, rs1560189257	5	0.58955

chr3:170021653..170021654	rs56925937	5	0.58955
chr3:170021654..170021655	rs1344345892	5	0.58955
chr3:170021655..170021656	rs1206259438	4	0.60906
chr3:170021656..170021657	rs1255717037, rs1270547107	4	0.60906
chr3:170021657..170021658	rs951067653	4	0.60906
chr3:170021658..170021659	rs1463878220	4	0.60906
chr3:170021668..170021669	rs568000356	3a	0.82517
chr3:170021670..170021671	rs1182699848	3a	0.82517
chr3:170021671..170021672	rs1365604920	3a	0.3231
chr3:170021676..170021677	rs745700299	3a	1
chr3:170021677..170021678	rs558389000	3a	0.45656
chr3:170021680..170021681	rs992029518	3a	0.82517
chr3:170021682..170021683	rs755779523	4	0.60906
chr3:170021687..170021688	rs1356520847	3a	0.79729
chr3:170021688..170021689	rs1413948577	3a	0.79729
chr3:170021690..170021691	rs969179238	3a	0.84868
chr3:170021693..170021694	rs750432403	4	0.60906
chr3:170021694..170021695	rs368393288	4	0.60906
chr3:170021698..170021699	rs140672226	4	0.60906
chr3:170021699..170021700	rs748768971	4	0.60906
chr3:170021700..170021701	rs1391607520	4	0.60906
chr3:170021702..170021703	rs1330395307	4	0.60906
chr3:170021708..170021709	rs1333489133	3a	0.47552
chr3:170021709..170021710	rs912520945	3a	0.99633
chr3:170021710..170021711	rs371185429	3a	0.59935
chr3:170021711..170021712	rs768171546	3a	0.57429
chr3:170021714..170021715	rs1349925093	3a	0.75204
chr3:170021715..170021716	rs1231865966	3a	0.35836
chr3:170021721..170021722	rs1376755572	2a	0.3145
chr3:170021726..170021727	rs1282640263	2a	0.90082
chr3:170021730..170021731	rs1447651774	2a	1
chr3:170021735..170021736	rs1279321988	3a	0.81951
chr3:170021743..170021744	rs1039907054	4	0.60906
chr3:170021744..170021745	rs1314874272	4	0.60906
chr3:170021757..170021758	rs898340139	4	0.60906
chr3:170021760..170021761	rs1360423170	4	0.60906
chr3:170021764..170021765	rs370987046	4	0.60906
chr3:170021765..170021766	rs1046832646	4	0.60906
chr3:170021766..170021767	rs1412149498	4	0.60906
chr3:170021768..170021769	rs540048559	3a	0.65177
chr3:170021771..170021772	rs1472926092	3a	0.52725
chr3:170021773..170021774	rs1252860113	3a	0.93733
chr3:170021774..170021775	rs1188317708	3a	0.61268

chr3:170021779..170021780	rs1436178791	3a	0.81114
chr3:170021783..170021784	rs1487706567	3a	0.88261
chr3:170021786..170021787	rs193000607	4	0.60906
chr3:170021791..170021792	rs1560189351	4	0.60906
chr3:170021792..170021793	rs1254225078	4	0.60906
chr3:170021803..170021804	rs1002535861	4	0.60906
chr3:170021811..170021812	rs1035323263	4	0.60906
chr3:170021814..170021815	rs1560189364	4	0.60906
chr3:170021820..170021821	rs958916292	3a	0.57256
chr3:170021822..170021823	rs145830938	3a	0.84623
chr3:170021840..170021841	rs1356363614	3a	0.61026
chr3:170021846..170021847	rs751579309	3a	0.57068
chr3:170021850..170021851	rs1462763870	3a	0.47043
chr3:170021862..170021863	rs138397245	3a	0.9531
chr3:170021876..170021877	rs1169304434	3a	0.70695
chr3:170021884..170021885	rs968937892	3a	0.4701
chr3:170021892..170021893	rs977896518	3a	0.49259
chr3:170021898..170021899	rs1455815396	4	0.60906
chr3:170021901..170021902	rs1348435276	4	0.60906
chr3:170021918..170021919	rs1164558204	3a	0.88155
chr3:170021942..170021943	rs1405545817	4	0.60906
chr3:170021943..170021944	rs115785967	4	0.60906
chr3:170021946..170021947	rs1168146533	3a	0.32979
chr3:170021952..170021953	rs954691371	3a	0.69529
chr3:170021958..170021959	rs1263347248	3a	0.85537
chr3:170021978..170021979	rs1188143463	4	0.60906
chr3:170021991..170021992	rs184700680	3a	0.85988
chr3:170021993..170021994	rs1245862260	3a	0.24403
chr3:170021995..170021996	rs1221999628	3a	0.94867
chr3:170022004..170022005	rs910562617	5	0.13454
chr3:170022012..170022013	rs1373170439	5	0.13454
chr3:170022014..170022015	rs1275275009	5	0.13454
chr3:170022015..170022016	rs771465377	5	0.13454
chr3:170022020..170022021	rs188094061	5	0.44483
chr3:170022021..170022022	rs1317308467	5	0.34168
chr3:170022037..170022038	rs975373691	6	0
chr3:170022040..170022041	rs919733880	6	0.34955
chr3:170022048..170022049	rs1293513232	7	0.18412
chr3:170022051..170022052	rs1442063207	7	0.18412
chr3:170022060..170022061	rs374193749	6	0.72948
chr3:170022071..170022072	rs1328855756	6	0.25986
chr3:170022073..170022074	rs1047252552	6	0
chr3:170022079..170022080	rs1316263566	6	0.17931
chr3:170022087..170022088	rs1431000408	7	0.18412

Table S3. Location, alleles, miR ID, miR site and function of 3′ UTR from polymiRTS analysis.

Location	dbSNP ID	Wobble base pair	Ancestral Allele	Allele	miR ID	Conservation	miRSite	Function Class	Exp Support	context+ score change
170020936	rs145216751	Y	A	A	hsa-miR-4802-3p	2	CCATGTAttctac	D	N	-0.169
					hsa-miR-942-3p	2	CCATGTAttctac	D	N	-0.167
				G	hsa-miR-4643	2	cCATGTGTtctac	C	N	-0.196
					hsa-miR-4717-3p	2	CCATGTGttctac	C	N	-0.26
					hsa-miR-4773	2	ccatgTGTTCTAc	C	N	-0.071
170020976	rs187988898	N	T	T	hsa-miR-103a-3p	0	actTGCTGCAagc	N	N	-0.172
					hsa-miR-107	0	actTGCTGCAagc	N	N	-0.172
					hsa-miR-1301-3p	0	acttGCTGCAAgc	N	N	-0.171
					hsa-miR-4327	0	acttgcTGCAAGC	N	N	-0.171
					hsa-miR-5047	0	acttGCTGCAAgc	N	N	-0.181
					hsa-miR-7156-3p	0	acttGCTGCAAgc	N	N	-0.165
				C	hsa-miR-6820-5p	0	actTGCCGCAagc	C	N	-0.266
170021160	rs71277165	Y	G	G	hsa-miR-1245b-5p	2	taaAAGGCCTAca	D	N	-0.468
					hsa-miR-3142	2	taaAAGGCCTAca	D	N	-0.478
					hsa-miR-6806-5p	2	taaaagGCCTACA	D	N	-0.208
170021245	rs190906216	N	C	C	hsa-miR-1271-3p	6	aAGGCACAtagtg	D	N	-0.173
					hsa-miR-455-5p	3	aaGGCACATAgtg	D	N	-0.393
					hsa-miR-550a-3-5p	6	aAGGCACAtagtg	D	N	-0.167
					hsa-miR-550a-5p	6	aAGGCACAtagtg	D	N	-0.167
					hsa-miR-550b-2-5p	6	aAGGCACAtagtg	D	N	-0.253
				A	hsa-miR-3152-5p	6	aAGGCAAAtagtg	C	N	-0.143
					hsa-miR-4774-3p	6	aAGGCAAAtagtg	C	N	-0.144
170021257	rs183165714	Y	A	A	hsa-miR-3171	3	gggatTACATCAt	D	N	-0.093
					hsa-miR-380-3p	4	gggATTACATcat	D	N	-0.015
				G	hsa-miR-1322	8	gggattGCATCAT	C	N	-0.243
170021396	rs3903277	Y	A	A	hsa-miR-3140-5p	9	aaATTCAGGtgta	D	N	-0.225
					hsa-miR-3622a-3p	13	aaatTCAGGTGta	D	N	-0.285
					hsa-miR-3622b-3p	13	aaatTCAGGTGta	D	N	-0.248
					hsa-miR-4680-3p	7	aAATTCAGgtgta	D	N	-0.116
170021450	rs115170199	N	C	C	hsa-miR-6882-3p	2	AGGCAGCAgttat	D	N	-0.413

				G	hsa-miR-1976	2	agGCAGGAGttat	C	N	-0.234
					hsa-miR-4722-3p	2	aGGCAGGAgttat	C	N	-0.174
					hsa-miR-4764-3p	10	aggcaGGAGTTAt	C	N	-0.17
					hsa-miR-6727-3p	2	aGGCAGGAgttat	C	N	-0.174
					hsa-miR-6747-3p	2	AGGCAGGAgttat	C	N	-0.382
				C	hsa-miR-32-3p	8	cagttaCTAAATT	C	N	No Change
					hsa-miR-802	6	caGTTACTAaatt	C	N	-0.046
				T	hsa-miR-3606-3p	2	GAAATTTggctgg	C	N	0.118
					hsa-miR-513a-3p	2	GAAATTTggctgg	C	N	0.099
					hsa-miR-513c-3p	2	GAAATTTggctgg	C	N	0.099
170021646	rs143395024	Y	A	A	hsa-miR-6872-3p	2	ctgGGCATGGtgg	D	N	-0.124
170021653	rs146869487	N	C	C	hsa-miR-1227-3p	2	tgGTGGCACAtac	D	N	-0.274
					hsa-miR-455-5p	2	tggtGGCACATAc	D	N	-0.202
					hsa-miR-4715-3p	2	tGGTGGCAcatac	D	N	-0.189
170021654	rs56925937	Y	A	A	hsa-miR-1227-3p	1	gGTGGCACAtacc	N	N	-0.274
					hsa-miR-455-5p	1	ggtGGCACATAcc	N	N	-0.202
					hsa-miR-4715-3p	2	GGTGGCAcatacc	D	N	-0.189
				G	hsa-miR-4787-3p	2	ggtGGCGCATAcc	C	N	-0.441
				G	hsa-miR-495-3p	10	cttTTTGTTAtga	C	N	0.069
					hsa-miR-5688	10	cttTTTGTTAtga	C	N	0.069
					hsa-miR-7-1-3p	10	cttTTTGTTAtga	C	N	0.062
					hsa-miR-7-2-3p	10	cttTTTGTTAtga	C	N	0.062
170021863	rs138397245	Y	A	A	hsa-miR-19a-5p	2	tGCAAAACtggca	D	N	-0.086
					hsa-miR-19b-1-5p	2	tGCAAAACtggca	D	N	-0.049
					hsa-miR-19b-2-5p	2	tGCAAAACtggca	D	N	-0.049
					hsa-miR-3680-3p	2	TGCAAAActggca	D	N	0.022
					hsa-miR-4328	7	tgcAAAACTGgca	D	N	0.067
					hsa-miR-450b-5p	2	TGCAAAActggca	D	N	-0.033
					hsa-miR-507	2	TGCAAAActggca	D	N	0.007
					hsa-miR-557	2	TGCAAAActggca	D	N	0.011
				G	hsa-miR-1202	5	tgcaaaGCTGGCA	C	N	-0.207
					hsa-miR-1251-3p	2	tGCAAAGCtggca	C	N	-0.137
					hsa-miR-3194-5p	5	tgcaaaGCTGGCA	C	N	-0.129
					hsa-miR-3972	5	tgcaaaGCTGGCA	C	N	-0.198
170021944	rs115785967	Y	A	A	hsa-miR-141-3p	1	aactgCAGTGTTA	N	N	-0.11
					hsa-miR-17-3p	1	aACTGCAGtgtta	N	N	-0.122
					hsa-miR-200a-3p	1	aactgCAGTGTTA	N	N	-0.089
					hsa-miR-4418	1	aaCTGCAGTgtta	N	N	-0.143

					hsa-miR-509-3-5p<br>hsa-miR-509-5p	1<br>1	aaCTGCAGTgtta<br>aaCTGCAGTgtta	N<br>N	N<br>N	-0.143<br>-0.143
				G	hsa-miR-21-3p<br>hsa-miR-3591-3p	2<br>2	aactgcGGTGTTA<br>aactgcGGTGTTA	C<br>C	N<br>N	-0.043<br>-0.043
170021992	rs184700680	Y	A	A	hsa-miR-4775<br>hsa-miR-587<br>hsa-miR-590-3p	5<br>4<br>7	atggAAAATTAct<br>ATGGAAAattact<br>atggAAAATTAct	D<br>D<br>D	N<br>N<br>N	0.187<br>0.029<br>0.2
				G	hsa-miR-141-5p	5	atGGAAGATtact	C	N	-0.081
170022021	rs188094061	N	C	C	hsa-miR-29b-1-5p	15	aaaAAACCAGAaa	D	N	-0.118
				A	hsa-miR-4652-3p<br>hsa-miR-4743-3p	16<br>16	aaaaaAACAGAAA<br>aaaaaaACAGAAA	C<br>C	N<br>N	-0.072<br>-0.018
170022166	rs141706029	N	T	T	hsa-miR-4427	10	catgTATTCAGtg	D	N	0.067
170022222	rs73036348	N	T	G	hsa-miR-5003-3p	0	gGAAAAGTAttta	C	N	0.076
				T	hsa-miR-4775<br>hsa-miR-5692a<br>hsa-miR-590-3p	1<br>1<br>1	ggAAAATTAttta<br>ggaaaATTATTTA<br>ggAAAATTAttta	N<br>N<br>N	N<br>N<br>N	0.169<br>0.219<br>0.171
170022285	rs114481051	Y	A	A	hsa-miR-577	1	TTATCTAtatgat	N	N	0.093
				G	hsa-miR-154-3p<br>hsa-miR-485-3p<br>hsa-miR-487a-3p<br>hsa-miR-539-3p<br>hsa-miR-5683	1<br>1<br>1<br>1<br>1	ttatctGTATGAT<br>ttatcTGTATGAt<br>ttatctGTATGAT<br>ttatcTGTATGAt<br>ttATCTGTAtgat	C<br>C<br>C<br>C<br>C	N<br>N<br>N<br>N<br>N	0.021<br>0.065<br>0.021<br>0.009<br>0.05
170022310	rs114316954	N	C	C	hsa-miR-29a-5p<br>hsa-miR-3192-3p<br>hsa-miR-3920<br>hsa-miR-4762-3p	4<br>4<br>4<br>4	tgAAATCAGAaaa<br>tgaaATCAGAAaa<br>tgaAATCAGAaaa<br>tgaaATCAGAAAa	D<br>D<br>D<br>D	N<br>N<br>N<br>N	0.088<br>0.02<br>0.043<br>0.046
				T	hsa-miR-4263<br>hsa-miR-4735-5p<br>hsa-miR-576-5p	6<br>4<br>4	tgaaaTTAGAAAa<br>tgAAATTAGAaaa<br>tgaaATTAGAAAa	C<br>C<br>C	N<br>N<br>N	0.095<br>0.19<br>0.106
170022328	rs116960340	Y	A	A	hsa-miR-24-1-5p<br>hsa-miR-24-2-5p<br>hsa-miR-664a-3p	2<br>2<br>3	GTAGGCAtgaata<br>GTAGGCAtgaata<br>gtaggcATGAATA	D<br>D<br>D	N<br>N<br>N	-0.082<br>-0.08<br>0.127
170022441	rs11707876	N	G	G	hsa-miR-146a-3p<br>hsa-miR-2682-3p<br>hsa-miR-4766-5p<br>hsa-miR-6781-3p	2<br>2<br>2<br>2	atTTCAGAGgcag<br>atttcAGAGGCAg<br>aTTTCAGAggcag<br>atttcAGAGGCAg	D<br>D<br>D<br>D	N<br>N<br>N<br>N	-0.015<br>-0.058<br>0.108<br>-0.051

				C	hsa-miR-214-5p<br>hsa-miR-6514-3p<br>hsa-miR-6811-3p	2<br>2<br>2	atttcACAGGCAg<br>atttcACAGGCAg<br>atttCACAGGCAg	C<br>C<br>C	N<br>N<br>N	-0.057<br>-0.03<br>-0.171
170022455	rs150111055	N	C	C	hsa-miR-3928-3p	1	GTTCCTCggatta	N	N	-0.024
				T	hsa-miR-6830-5p	1	gTTCCTTGgatta	C	N	0.014
170022495	rs2650220	Y	A	A	hsa-miR-5692a	9	cttATTATTTAaa	D	N	0.294
170022758	rs138385075	N	T	T	hsa-miR-4311<br>hsa-miR-651-3p	4<br>3	acTCTCTTTcctt<br>actctcTTTCCTT	D<br>D	N<br>N	0.03<br>0.016
				C	hsa-miR-4428<br>hsa-miR-4476<br>hsa-miR-4533<br>hsa-miR-5192<br>hsa-miR-6876-5p	4<br>2<br>2<br>3<br>2	acTCTCCTTcctt<br>actctCCTTCCTt<br>actcTCCTTCCtt<br>ACTCTCCttcctt<br>actctCCTTCCTt	C<br>C<br>C<br>C<br>C	N<br>N<br>N<br>N<br>N	-0.017<br>-0.108<br>-0.08<br>-0.146<br>-0.108
170022800	rs149238335	N	A	A	hsa-miR-3121-5p	5	acgtCAAAGGAaa	D	N	-0.027
				C	hsa-miR-1914-5p<br>hsa-miR-3074-5p<br>hsa-miR-7152-5p	5<br>6<br>6	acgtCACAGGAaa<br>acgtcaCAGGAAA<br>acgtcACAGGAAA	C<br>C<br>C	N<br>N<br>N	-0.081<br>-0.021<br>-0.074
170023009	rs192326883	N	C	C	hsa-miR-5571-3p<br>hsa-miR-6800-5p	3<br>7	taatcaCCTAGGA<br>taaTCACCTAgga	D<br>D	N<br>N	-0.196<br>-0.171
170023020	rs3914146	N	G	G	hsa-miR-497-3p	10	gaagtTGGTTTAa	D	N	-0.011
				T	hsa-miR-495-3p<br>hsa-miR-5688	8<br>8	gaaGTTTGTTtaa<br>gaaGTTTGTTtaa	C<br>C	N<br>N	-0.037<br>-0.037
170023041	rs3914145	Y	A	A	hsa-miR-4284<br>hsa-miR-4417<br>hsa-miR-4437<br>hsa-miR-4674	1<br>2<br>2<br>2	ctaGTGAGCCcac<br>ctagtgAGCCCAC<br>ctagTGAGCCCAc<br>ctagtGAGCCCAc	N<br>D<br>D<br>D	N<br>N<br>N<br>N	-0.15<br>-0.153<br>-0.25<br>-0.156
				G	hsa-miR-296-5p<br>hsa-miR-4301<br>hsa-miR-6724-5p<br>hsa-miR-6773-5p	2<br>1<br>2<br>2	ctagtGGGCCCAc<br>cTAGTGGGcccac<br>ctagtGGGCCCAc<br>ctagtGGGCCCAc	C<br>C<br>C<br>C	N<br>N<br>N<br>N	-0.154<br>-0.067<br>-0.208<br>-0.227
170023051	rs147435942	N	A	A	hsa-miR-18a-5p<br>hsa-miR-18b-5p<br>hsa-miR-4521<br>hsa-miR-4735-3p	5<br>5<br>6<br>5	CACCTTAgacttc<br>CACCTTAgacttc<br>caCCTTAGActtc<br>CACCTTAgacttc	D<br>D<br>D<br>D	N<br>N<br>N<br>N	-0.056<br>-0.056<br>-0.018<br>-0.042
				A	hsa-miR-548n	2	tagacTACTTTTg	C	N	0.134
170023064	rs1849047	Y	G	G	hsa-miR-4493<br>hsa-miR-5699-5p<br>hsa-miR-874-5p	2<br>3<br>2	ttttggGGCCTTC<br>ttTTGGGGCcttc<br>tttTGGGGCCttc	D<br>D<br>D	N<br>N<br>N	-0.112<br>-0.215<br>-0.246
				A	hsa-miR-519d-5p	2	tTTTGGAGcttc	C	N	-0.05

				A	hsa-miR-377-5p	1	gcculCAACCTCA	C	N	-0.199
					hsa-miR-4493	2	GCCTTCAacctca	C	N	-0.145
					hsa-miR-605-3p	2	GCCTTCAacctca	C	N	-0.077
					hsa-miR-6086	1	gccttCAACCTCA	C	N	-0.23
					hsa-miR-6126	2	GCCTTCAAcctca	C	N	-0.163
					hsa-miR-655-5p	5	gccttcAACCTCA	C	N	-0.056
					hsa-miR-6864-5p	2	gCCTTCAAcctca	C	N	-0.022
170023169	rs189382403	N	A	A	hsa-miR-6798-3p	3	GGGGTAAgcagtg	D	N	-0.165
170023396	rs9824427	N	C	C	hsa-miR-5700	3	gaaATGCATTAta	D	N	-0.287
					hsa-miR-586	4	gaAATGCATtata	D	N	-0.17
				T	hsa-miR-369-3p	11	gaaatGTATTATA	C	N	-0.18
					hsa-miR-374a-5p	11	gaaatgTATTATA	C	N	-0.096
					hsa-miR-374b-5p	11	gaaatgTATTATA	C	N	-0.096
					hsa-miR-374c-5p	9	gaaaTGTATTAta	C	N	-0.158
					hsa-miR-5692b	12	gaaatgTATTATA	C	N	-0.003
					hsa-miR-5692c	12	gaaatgTATTATA	C	N	-0.003
					hsa-miR-655-3p	9	gaaaTGTATTAta	C	N	-0.148
				A	hsa-miR-181a-5p	2	ggatTGAATGTta	C	N	-0.142
					hsa-miR-181b-5p	2	ggatTGAATGTta	C	N	-0.142
					hsa-miR-181c-5p	2	ggatTGAATGTta	C	N	-0.133
					hsa-miR-181d-5p	2	ggatTGAATGTta	C	N	-0.124
					hsa-miR-3143	2	ggattgAATGTTA	C	N	-0.092
					hsa-miR-4262	2	ggatTGAATGTta	C	N	-0.152
					hsa-miR-543	2	ggattGAATGTTA	C	N	-0.222
				C	hsa-miR-595	10	ggtACACTTActt	C	N	-0.074
							aataaaatgtATATTGAg	O	N	-0.035
							aataaaatgtATATTGAg	O	N	-0.024

Table S4. Functionally significant 3′ UTR SNP analysis by miRNASNP.

SNP ID	Ref/Alt	Region	Ref Frequency	LD	Gain		Loss	
rs145216751	A/G	3'UTR	0.9999/0.0001	-	5	hsa-miR-4773<br>hsa-miR-4717-3p<br>hsa-miR-5687<br>hsa-miR-4789-3p<br>hsa-miR-4643	1	hsa-miR-4666a-5p
rs71277165	G/A<br>G/T	3'UTR	0.9805/0.0195<br>9805	Yes<br>Yes	1<br>6	hsa-miR-4759<br>hsa-miR-3117-3p<br>hsa-miR-8083<br>hsa-miR-3169<br>hsa-miR-5003-3p<br>hsa-miR-6830-3p<br>hsa-miR-8065	5<br><br><br><br><br>5	hsa-miR-1245b-5p<br>hsa-miR-5704<br>hsa-miR-1295a<br>hsa-miR-3142<br>hsa-miR-6806-5p<br>hsa-miR-1245b-5p<br>hsa-miR-5704<br>hsa-miR-1295a<br>hsa-miR-3142<br>hsa-miR-6806-5p
rs190906216	C/A	3'UTR	-	-	0		5	hsa-miR-455-5p<br>hsa-miR-1271-3p<br>hsa-miR-550a-5p<br>hsa-miR-550a-3-5p<br>hsa-miR-550b-2-5p
rs183165714	A/G	3'UTR	0.998/0.0020	Yes	1	hsa-miR-106a-3p	6	hsa-miR-411-3p<br>hsa-miR-379-3p<br>hsa-miR-3171<br>hsa-miR-4495<br>hsa-miR-380-3p<br>hsa-miR-380-3p<br>hsa-miR-2054
rs3903277	A/G	3'UTR	-	-	0		9	hsa-miR-4680-3p<br>hsa-miR-183-3p<br>hsa-miR-6765-3p<br>hsa-miR-3622a-3p<br>hsa-miR-4427<br>hsa-miR-3622b-3p<br>hsa-miR-4452<br>hsa-miR-5187-3p<br>hsa-miR-3140-5p
rs115170199	C/G	3'UTR	0.9699/0.0301	Yes	0		3	hsa-miR-93-3p

								hsa-miR-3692-5p<br>hsa-miR-6882-3p
rs3896073	T/C	3'UTR	-	-	1	hsa-miR-802	0	
rs187434851	C/T	3'UTR	0.9999/0.0001	-	4	hsa-miR-513a-3p<br>hsa-miR-4691-3p<br>hsa-miR-3606-3p<br>hsa-miR-513c-3p	0	
rs143395024	A/C<br>A/G	3'UTR	0.9894/-<br>0.9894/0.0105	Yes<br>Yes	1<br>0	hsa-miR-4747-3p	2<br>2	hsa-miR-7153-3p<br>hsa-miR-6872-3p
rs146869487	C/T	3'UTR	0.9746/0.0254	Yes	2	hsa-miR-12129<br>hsa-miR-3924	6	hsa-miR-4715-3p<br>hsa-miR-455-5p<br>hsa-miR-1227-3p<br>hsa-miR-550b-2-5p<br>hsa-miR-12121<br>hsa-miR-3064-3p
rs56925937	A/G	3'UTR	1/-	-	1	hsa-miR-6807-5p	7	hsa-miR-4715-3p<br>hsa-miR-3064-3p<br>hsa-miR-12121<br>hsa-miR-567<br>hsa-miR-550b-2-5p<br>hsa-miR-1227-3p<br>hsa-miR-455-5p
rs145830938	A/G	3'UTR	0.9987/0.0013	-	4	hsa-miR-495-3p<br>hsa-miR-5688	4	hsa-miR-5692b<br>hsa-miR-369-3p<br>hsa-miR-3163<br>hsa-miR-5692c
rs115785967	A/G	3'UTR	0.9805/0.0195	Yes	3	hsa-miR-6886-5p<br>hsa-miR-21-3p<br>hsa-miR-122b-3p	14	hsa-miR-1205<br>hsa-miR-6890-3p<br>hsa-miR-17-3p<br>hsa-miR-200a-3p<br>hsa-miR-1184<br>hsa-miR-509-3-5p<br>hsa-miR-3158-5p<br>hsa-miR-509-5p<br>hsa-miR-4418<br>hsa-miR-141-3p<br>hsa-miR-217-5p<br>hsa-miR-6807-3p<br>hsa-miR-6757-3p<br>hsa-miR-10394-5p

rs184700680	A/G	3'UTR	0.9981/0.0019	-	1	hsa-miR-141-5p	2	hsa-miR-590-3p<br>hsa-miR-4775
rs188094061	C/A	3'UTR	0.9962/0.0038	Yes	0		2	hsa-miR-29b-2-5p<br>hsa-miR-29b-1-5p
rs141706029	T/G	3'UTR	0.9966/0.0034	Yes	2	hsa-miR-888-3p<br>hsa-miR-345-5p	5	hsa-miR-5585-3p<br>hsa-miR-4427<br>hsa-miR-4680-3p<br>hsa-miR-4724-5p<br>hsa-miR-5187-3p
rs73036348	G/T	3'UTR	0.9028/0.0972	Yes	2	hsa-miR-4775<br>hsa-miR-590-3p	6	hsa-miR-8084<br>hsa-miR-5003p<br>hsa-miR-429<br>hsa-miR-200b-3p<br>hsa-miR-200c-3p<br>hsa-miR-627-3p
rs114481051	A/G	3'UTR	0.9923/0.0077	Yes	3	hsa-miR-5683<br>hsa-miR-485-3p<br>hsa-miR-539-3p	2	hsa-miR-10397-3p<br>hsa-miR-5007-3p
rs114316954	C/T	3'UTR	0.9804/0.0196	Yes	4	hsa-miR-4735-5p<br>hsa-miR-4775<br>hsa-miR-576-5p<br>hsa-miR-4263	4	
rs116960340	A/G	3'UTR	0.9982/0.0018	Yes	0	-	2	hsa-miR-664a-3p<br>hsa-miR-532-5p
rs11707876	G/C		-	-	8	hsa-miR- 4677-3p<br>hsa-miR- 3611<br>hsa-miR- 6811-3p<br>hsa-miR- 6894-3p<br>hsa-miR- 4679<br>hsa-miR- 6514-3p<br>hsa-miR- 1914-5p<br>hsa-miR- 214-5p	9	hsa-miR- 301a-5p<br>hsa-miR- 6781-3p<br>hsa-miR- 4653-5p<br>hsa-miR- 4766-5p<br>hsa-miR- 6836-3p<br>hsa-miR- 2682-3p<br>hsa-miR- 146a-3p<br>hsa-miR- 3921<br>hsa-miR- 301b-5p
rs150111055	C/T		0.9979/0.0021	Yes	2	hsa-miR-651-3p<br>hsa-miR-6830-5p	5	hsa-miR-3928-3p<br>hsa-miR-611<br>hsa-miR-3131<br>hsa-miR-151a-5p<br>hsa-miR-151b
rs2650220	A/G		0.8721/0.1279	Yes	2	hsa-miR-338-5p<br>hsa-miR-95-5p	4	hsa-miR-5692b<br>hsa-miR-369-3p

								hsa-miR-5692a<br>hsa-miR-5692c
rs138385075	T/C		0.9956/0.0044	-	13	hsa-miR-3202<br>hsa-miR-185-5p<br>hsa-miR-6891-5p<br>hsa-miR-12118<br>hsa-miR-4644<br>hsa-miR-6876-5p<br>hsa-miR-5192<br>hsa-miR-4533<br>hsa-miR-4476<br>hsa-miR-4306<br>hsa-miR-3173-3p<br>hsa-miR-6888-5p<br>hsa-miR-4428	7	hsa-miR-4311<br>hsa-miR-4496<br>hsa-miR-12124<br>hsa-miR-6856-5p<br>hsa-miR-6758-5p<br>hsa-miR-583<br>hsa-miR-1276
rs149238335	A/C		0.9999/0.0001	-	6	hsa-miR-1914-5p<br>hsa-miR-7152-5p<br>hsa-miR-6808-3p<br>hsa-miR-4679<br>hsa-miR-4677-3p<br>hsa-miR-3074-5p	1	hsa-miR-3121-5p
rs192326883	C/T		0.9997/0.0003	Yes	1	hsa-miR-3682-3p	2	hsa-miR-6800-5p<br>hsa-miR-6802-5p
rs3914146	G/T		-	-	4	hsa-miR-5688<br>hsa-miR-7-1-3p<br>hsa-miR-7-2-3p<br>hsa-miR-495-3p	1	hsa-miR-497-3p
rs3914145	A/G		-	-	6	hsa-miR-4301<br>hsa-miR-3136-3p<br>hsa-miR-6773-5p<br>hsa-miR-296-5p<br>hsa-miR-7155-3p<br>hsa-miR-6724-5p	8	hsa-miR-3620-5p<br>hsa-miR-4284<br>hsa-miR-10400-3p<br>hsa-miR-1304-3p<br>hsa-miR-4437<br>hsa-miR-1587<br>hsa-miR-4674<br>hsa-miR-24-3p
rs147435942	A/T		-	-	2	hsa-miR-548m<br>hsa-miR-12135	3	hsa-miR-1264<br>hsa-miR-3184-3p<br>hsa-miR-4521
rs183522430	T/A		0.9997/0.0003	-	3	hsa-miR-548j-5p<br>hsa-miR-548k	4	hsa-miR-3184-3p<br>hsa-miR-186-5p

						hsa-miR-548c-5p		hsa-miR-3133<br>hsa-miR-1264
rs1849047	G/A		-	-	3	hsa-miR-10398-5p<br>hsa-miR-519d-5p<br>hsa-miR-5695	8	hsa-miR-4758-3p<br>hsa-miR-4753-5p<br>hsa-miR-5699-5p<br>hsa-miR-6793-3p<br>hsa-miR-874-5p<br>hsa-miR-186-3<br>hsa-miR-4747-3p<br>hsa-miR-3189-5p
rs1849046	G/A		1/-	-	7	hsa-miR-6086<br>hsa-miR-655-5p<br>hsa-miR-380-5p<br>hsa-miR-6126<br>hsa-miR-6864-5p<br>hsa-miR-377-5p<br>hsa-miR-563	2	hsa-miR-431-3p<br>hsa-miR-431-3p
rs189382403	A/C		0.9997/0.0003	yes	2	hsa-miR-126-3p<br>hsa-miR-4520-5p	0	
rs9824427	C/A		0.9876/-	yes	5	hsa-miR-664b-3p<br>hsa-miR-579-3p<br>hsa-miR-5692c<br>hsa-miR-369-3p<br>hsa-miR-4760-3p	5	hsa-miR-5700<br>hsa-miR-586<br>hsa-miR-33a-5p<br>hsa-miR-155-5p<br>hsa-miR-33b-5p
rs192431985	G/A		0.9999/0.0001	-	7	hsa-miR-181c-5p<br>hsa-miR-181a-5p<br>hsa-miR-181b-5p<br>hsa-miR-543<br>hsa-miR-4272<br>hsa-miR-181d-5p<br>hsa-miR-4262	0	
rs184503621	T/C		0.999/0.0010	-	3	hsa-miR-3177-5p<br>hsa-miR-595<br>hsa-miR-4280	1	hsa-miR-12129

Table S5. Functionally significant 3′ UTR SNP analysis by MicroSNIper.

SNP	Range	MicroRNA
rs190906216	chr3:170020916-170023770	hsa-miR-455-5p<br>hsa-miR-550b-2-5p<br>hsa-miR-1282<br>hsa-miR-605
rs187988898	chr3:170020916-170023770	hsa-miR-3944-5p<br>hsa-miR-4327<br>hsa-miR-3120-3p<br>hsa-miR-761<br>hsa-miR-4637<br>hsa-miR-5047<br>hsa-miR-508-3p<br>hsa-miR-4673<br>hsa-miR-4327
rs193000607	chr3:170020916-170023770	hsa-miR-5002-5p<br>hsa-miR-4699-5p
rs146869487	chr3:170020916-170023770	hsa-miR-1227-3p<br>hsa-miR-3159<br>hsa-miR-455-5p<br>hsa-miR-4715-3p<br>hsa-miR-5095<br>hsa-miR-220b-2-5p<br>hsa-miR-600
rs140672226	chr3:170020916-170023770	hsa-miR-23b-5p<br>hsa-miR-498<br>hsa-miR-4670-5p<br>hsa-miR-661<br>hsa-miR-23b-5p
rs187434851	chr3:170020916-170023770	hsa-miR-501-3p<br>hsa-miR-4530
rs71277165	chr3:170020916-170023770	hsa-miR-3650<br>hsa-miR-3142<br>hsa-miR-3137<br>hsa-miR-3650<br>hsa-miR-1183<br>hsa-miR-3137
rs183165714	chr3:170020916-170023770	hsa-miR-3689b-3p

		hsa-miR-3689c<br>hsa-miR-3689a-3p<br>hsa-miR-301b
rs115170199	chr3:170020916-170023770	hsa-miR-532-5p<br>hsa-miR-1976<br>hsa-miR-4764-3p
rs138397245	chr3:170020916-170023770	hsa-miR-19a-5p<br>hsa-miR-19b-2-5p<br>hsa-miR-548d-3p<br>hsa-miR-19b-1-5p<br>hsa-miR-548t-3p<br>hsa-miR-3194<br>hsa-miR-4267<br>hsa-miR-3972<br>hsa-miR-1202
rs145830938		hsa-miR-5688<br>hsa-miR-3143
rs141706029	chr3:170020916-170023770	hsa-miR-655<br>hsa-miR-4666a-5p<br>hsa-miR-4427<br>hsa-miR-5585-3p<br>hsa-miR-181a-2-3p<br>hsa-miR-888-3p<br>hsa-miR-6082
rs115785967	chr3:170020916-170023770	hsa-miR-4418<br>hsa-miR-200a-3p<br>hsa-miR-141-3p<br>hsa-miR-17-3p<br>hsa-miR-509-5p<br>hsa-miR-5011-5p<br>hsa-miR-3158-5p<br>hsa-miR-1273g-3p<br>hsa-miR-509-3-5p<br>hsa-miR-21-3p
rs184700680	chr3:170020916-170023770	hsa-miR-590-3p<br>hsa-let-7f-3p<br>hsa-miR-4796-5p<br>hsa-miR-98-3p<br>hsa-miR-1185-2-3p<br>hsa-miR-141-5p<br>hsa-miR-98-3p

		hsa-miR-1911-5p
rs188094061	chr3:170020916-170023770	hsa-miR-4782-5p<br>hsa-miR-3606-3p<br>hsa-miR-4653-3p
rs114316954	chr3:170020916-170023770	hsa-miR-29a-5p<br>hsa-miR-205-3p<br>hsa-miR-4762-3p<br>hsa-miR-576-5p<br>hsa-miR-513a-3p<br>hsa-miR-513c-3p
rs114481051	chr3:170020916-170023770	hsa-miR-487a<br>hsa-miR-154-3p<br>hsa-miR-4705<br>hsa-miR-487a<br>hsa-miR-488-5p<br>hsa-miR-154-3p<br>hsa-miR-539-3p<br>hsa-miR-485-3p
rs11707876	chr3:170020916-170023770	hsa-miR-146a-3p<br>hsa-miR-5001-3p<br>hsa-miR-3152-3p<br>hsa-miR-4714-5p<br>hsa-miR4757-5p<br>hsa-miR-326<br>hsa-miR-5684<br>hsa-miR-214-5p<br>has-miR-550a-3-5p<br>hsa-miR-5008-3p<br>hsa-miR-661<br>hsa-miR-3116
rs116960340	chr3:170020916-170023770	hsa-miR-24-2-5p<br>hsa-miR-24-1-5p<br>hsa-miR-3657<br>hsa-miR-4760-3p
rs2650220	chr3:170020916-170023770	hsa-miR-5692a<br>hsa-miR-369-3p<br>hsa-miR-338-5p
rs150111055	chr3:170020916-170023770	hsa-miR-4454<br>hsa-miR-3928<br>hsa-miR-151b<br>hsa-miR-3125

		hsa-miR-151a-5p<br>hsa-miR-766-5p<br>hsa-miR-615-3p<br>hsa-miR-500a-5p<br>hsa-miR-3914
rs3914145	chr3:170020916-170023770	hsa-miR-4296<br>hsa-miR-4265<br>hsa-miR-4322<br>hsa-miR-4284<br>hsa-miR-4417<br>hsa-miR-4437<br>hsa-miR-4283<br>hsa-miR-1178-3p<br>hsa-miR-4674<br>hsa-miR-378g
rs138385075	chr3:170020916-170023770	hsa-miR-4311<br>hsa-miR-1252<br>hsa-miR-5192<br>hsa-miR-5093<br>hsa-miR-4428<br>has-miR-3676-5p
rs192326883	chr3:170020916-170023770	hsa-miR-5571-3p<br>hsa-miR-331-5p<br>hsa-miR-384<br>hsa-miR-4777-5p<br>hsa-miR-1245b-3p<br>hsa-miR-4770<br>hsa-miR-4305<br>hsa-miR-143-3
rs149238335	chr3:170020916-170023770	hsa-miR-501-5p<br>hsa-miR-543<br>hsa-miR-4501<br>hsa-miR-5008-3p<br>hsa-miR-3074-5p<br>hsa-miR-30e-5p<br>hsa-miR-3164
rs3914146	chr3:170020916-170023770	hsa-miR-6715a-3p<br>hsa-miR-5688<br>hsa-miR-7-1-3p
rs112129292	chr3:170020916-170023770	hsa-miR-3607-5p

		hsa-miR-4422<br>hsa-miR-4666b
rs9824427	chr3:170020916-170023770	hsa-miR-5700<br>hsa-miR-369-3p<br>hsa-miR-374b-5p<br>hsa-miR-374c-5p<br>hsa-miR-543<br>hsa-miR-4666a-5p<br>hsa-miR-340-5p<br>hsa-miR-374a-5p<br>hsa-miR-586<br>hsa-miR-502-3p
rs75142839	chr3:170020916-170023770	hsa-miR-3136<br>hsa-miR-329<br>hsa-miR-3941<br>hsa-miR-3591-3p<br>hsa-miR-21-3p<br>hsa-miR-767-5p
rs183522430	chr3:170020916-170023770	hsa-miR-3653<br>hsa-miR-548ax<br>hsa-miR-548n<br>hsa-miR-5197-3p<br>hsa-miR-548g-5p<br>hsa-miR-548x-5p<br>hsa-miR-548aj-5p<br>hsa-miR-548av-5p
rs1849046	chr3:170020916-170023770	hsa-miR-1294<br>hsa-miR-4710<br>hsa-miR-1224-5p<br>hsa-miR-431-3p<br>hsa-miR-6126<br>hsa-miR-1294<br>hsa-miR-563<br>hsa-miR-548ah<br>hsa-miR-6086<br>hsa-miR-4709-3p<br>hsa-miR-323b-5p
rs147435942	chr3:170020916-170023770	hsa-miR-513-5p<br>hsa-miR-18b-5p<br>hsa-miR-4735-3p<br>hsa-miR-3974

		hsa-miR-4468<br>hsa-miR-527<br>hsa-miR-518a-5p<br>hsa-miR-4422
rs189382403	chr3:170020916-170023770	hsa-miR-1208<br>hsa-miR-4720-3p<br>hsa-miR-4804-3p<br>hsa-miR-93-3<br>hsa-miR-4420
rs184503621	chr3:170020916-170023770	hsa-miR-545-5p<br>hsa-miR-3668<br>hsa-miR-3658<br>hsa-miR-6513-3p<br>hsa-miR-4280<br>hsa-miR-595<br>hsa-miR-3177<br>hsa-miR-302b-3p
rs192431985	chr3:170020916-170023770	hsa-miR-499-3p<br>hsa-miR-4793-5p<br>hsa-miR-181c-5p<br>hsa-miR-181a-5p<br>hsa-miR-181d<br>hsa-miR-4262<br>hsa-miR-4272<br>hsa-miR-1179<br>hsa-miR-4699<br>hsa-miR-3134<br>hsa-miR-4705
rs143709842	chr3:170020916-170023770	hsa-miR-217<br>hsa-miR-19b-3p<br>hsa-miR-298<br>hsa-miR-646<br>hsa-miR-320d<br>hsa-miR-320c<br>hsa-miR-4429

Table S6. Association of putative miRNA with UTRs SNPs predicted through PolymiRTS, MiRNASNP and MicroSNIper.

SNP ID	Location	PolymiRTS	MiRNASNP	MicroSNIper

rs71277165	170021160	hsa-miR-1245b-5p<br>**hsa-miR-3142**<br>hsa-miR-6806-5p	hsa-miR-1245b-5p<br>hsa-miR-5704<br>hsa-miR-1295a<br>**hsa-miR-3142**<br>hsa-miR-6806-5p<br>hsa-miR-1245b-5p<br>hsa-miR-5704<br>hsa-miR-1295a<br>hsa-miR-3142<br>hsa-miR-6806-5p<br>hsa-miR-4759<br>hsa-miR-3117-3p<br>hsa-miR-8083<br>hsa-miR-3169<br>hsa-miR-5003-3p<br>hsa-miR-6830-3p<br>hsa-miR-8065	hsa-miR-3650<br>**hsa-miR-3142**<br>hsa-miR-3137<br>hsa-miR-3650<br>hsa-miR-1183<br>hsa-miR-3137
rs190906216	170021245	hsa-miR-1271-3p<br>**hsa-miR-455-5p**<br>hsa-miR-550a-3-5p<br>hsa-miR-550a-5p<br>hsa-miR-550b-2-5p<br>hsa-miR-3152-5p<br>hsa-miR-4774-3p	**hsa-miR-455-5p**<br>hsa-miR-1271-3p<br>hsa-miR-550a-5p<br>hsa-miR-550a-3-5p<br>hsa-miR-550b-2-5p	**hsa-miR-455-5p**<br>hsa-miR-550b-2-5p<br>hsa-miR-1282<br>hsa-miR-605
rs183165714	170021257	hsa-miR-3171<br>hsa-miR-380-3p<br>hsa-miR-1322	hsa-miR-106a-3p<br>hsa-miR-411-3p<br>hsa-miR-379-3p<br>hsa-miR-3171<br>hsa-miR-4495<br>hsa-miR-380-3p<br>hsa-miR-380-3p<br>hsa-miR-2054	hsa-miR-3689b-3p<br>hsa-miR-3689c<br>hsa-miR-3689a-3p<br>hsa-miR-301b
rs115170199	170021450	**hsa-miR-6882-3p**<br>hsa-miR-1976<br>hsa-miR-4722-3p	hsa-miR-93-3p<br>hsa-miR-3692-5p<br>**hsa-miR-6882-3p**	hsa-miR-532-5p<br>hsa-miR-1976<br>hsa-miR-4764-3p<br>**hsa-miR-6882-3p**

		hsa-miR-4764-3p<br>hsa-miR-6727-3p<br>hsa-miR-6747-3p		
rs146869487	170021653	**hsa-miR-1227-3p**<br>hsa-miR-455-5p<br>hsa-miR-4715-3p	hsa-miR-12129<br>hsa-miR-3924<br>hsa-miR-4715-3p<br>hsa-miR-455-5p<br>**hsa-miR-1227-3p**<br>hsa-miR-550b-2-5p<br>hsa-miR-12121<br>hsa-miR-3064-3p	**hsa-miR-1227-3p**<br>hsa-miR-3159<br>hsa-miR-455-5p<br>hsa-miR-4715-3p<br>hsa-miR-5095<br>hsa-miR-220b-2-5p<br>hsa-miR-600
rs115785967	170021944	**hsa-miR-141-3p**<br>hsa-miR-17-3p<br>hsa-miR-200a-3p<br>**hsa-miR-4418**<br>hsa-miR-509-3-5p<br>**hsa-miR-509-5p**	hsa-miR-6886-5p<br>hsa-miR-21-3p<br>hsa-miR-6890-3p<br>hsa-miR-17-3p<br>hsa-miR-200a-3p<br>**hsa-miR-1184**<br>hsa-miR-509-3-5p<br>hsa-miR-3158-5p<br>**hsa-miR-509-5p**<br>hsa-miR-4418<br>**hsa-miR-141-3p**<br>hsa-miR-217-5p	**hsa-miR-4418**<br>hsa-miR-200a-3p<br>**hsa-miR-141-3p**<br>hsa-miR-17-3p<br>**hsa-miR-509-5p**<br>hsa-miR-5011-5p<br>hsa-miR-3158-5p<br>hsa-miR-1273g-3p<br>hsa-miR-509-3-5p<br>hsa-miR-21-3p
rs184700680	170021992	hsa-miR-4775<br>hsa-miR-587<br>**hsa-miR-590-3p**<br>**hsa-miR-141-5p**	**hsa-miR-141-5p**<br>**hsa-miR-590-3p**<br>hsa-miR-4775	**hsa-miR-590-3p**<br>hsa-miR-98-3p<br>hsa-miR-1185-2-3p<br>**hsa-miR-141-5p**<br>hsa-miR-98-3p<br>hsa-miR-1911-5p
rs188094061	170022021	hsa-miR-29b-1-5p<br>hsa-miR-4652-3p<br>hsa-miR-4743-3p	hsa-miR-29b-2-5p<br>hsa-miR-29b-1-5p	hsa-miR-4782-5p<br>hsa-miR-3606-3p<br>hsa-miR-4653-3p
rs141706029	170022166	**hsa-miR-4427**	hsa-miR-888-3p<br>hsa-miR-345-5p<br>hsa-miR-5585-3p<br>**hsa-miR-4427**<br>hsa-miR-4680-3p<br>hsa-miR-4724-5p	hsa-miR-655<br>hsa-miR-4666a-5p<br>**hsa-miR-4427**<br>hsa-miR-5585-3p<br>hsa-miR-181a-2-3p<br>hsa-miR-888-3p

			hsa-miR-5187-3p	hsa-miR-6082
rs114481051	170022285	hsa-miR-577<br>hsa-miR-154-3p<br>hsa-miR-485-3p<br>hsa-miR-487a-3p<br>hsa-miR-539-3p<br>hsa-miR-5683	hsa-miR-5683<br>hsa-miR-485-3p<br>hsa-miR-539-3p	hsa-miR-10397-3p<br>hsa-miR-5007-3p
rs114316954	170022310	hsa-miR-29a-5p<br>hsa-miR-3192-3p<br>hsa-miR-3920<br>hsa-miR-4762-3p<br>hsa-miR-4263<br>**hsa-miR-4735-5p**<br>hsa-miR-576-5p	**hsa-miR-4735-5p**<br>hsa-miR-4775<br>hsa-miR-576-5p<br>hsa-miR-4263	hsa-miR-29a<br>hsa-miR-3192-3p<br>hsa-miR-3920<br>hsa-miR-4762-3p<br>**hsa-miR-4735-5p**
rs116960340	170022328	hsa-miR-24-1-5p<br>hsa-miR-24-2-5p<br>hsa-miR-664a-3p	hsa-miR-664a-3p<br>hsa-miR-532-5p	hsa-miR-24-2-5p<br>hsa-miR-24-1-5p<br>hsa-miR-3657<br>hsa-miR-4760-3p
rs11707876	170022441	**hsa-miR-146a-3p**<br>hsa-miR-2682-3p	hsa-miR- 4677-3p<br>hsa-miR- 3611<br>hsa-miR- 6811-3p<br>hsa-miR- 6894-3p<br>hsa-miR- 4679<br>hsa-miR- 6514-3p<br>hsa-miR- 1914-5p<br>hsa-miR- 214-5p<br>hsa-miR- 301a-5p<br>hsa-miR- 6781-3p<br>hsa-miR- 4653-5p<br>hsa-miR- 4766-5p<br>hsa-miR- 6836-3p<br>hsa-miR- 2682-3p<br>**hsa-miR- 146a-3p**<br>hsa-miR- 3921<br>hsa-miR- 301b-5p	**hsa-miR-146a-3p**<br>hsa-miR-5001-3p<br>hsa-miR-3152-3p<br>hsa-miR-4714-5p<br>hsa-miR4757-5p<br>hsa-miR-326<br>hsa-miR-5684<br>hsa-miR-214-5p<br>has-miR-550a-3-5p<br>hsa-miR-5008-3p<br>hsa-miR-661<br>hsa-miR-3116
rs150111055	170022455	**hsa-miR-3928-3p**<br>hsa-miR-6830-5p	hsa-miR-651-3p<br>hsa-miR-6830-5p<br>**hsa-miR-3928-3p**	hsa-miR-4454<br>**hsa-miR-3928-3p**<br>hsa-miR-151b

			hsa-miR-611<br>hsa-miR-3131<br>hsa-miR-151a-5p<br>hsa-miR-151b	hsa-miR-3125<br>hsa-miR-151a-5p<br>hsa-miR-766-5p
rs2650220	170022<br>495	**hsa-miR-5692a**	hsa-miR-338-5p<br>hsa-miR-95-5p<br>hsa-miR-5692b<br>hsa-miR-369-3p<br>**hsa-miR-5692a**	**hsa-miR-5692a**<br>hsa-miR-369-3p<br>hsa-miR-338-5p
rs138385075	170022758	**hsa-miR-4311**<br>hsa-miR-651-3p<br>hsa-miR-4428<br>hsa-miR-4476<br>hsa-miR-4533<br>hsa-miR-5192<br>hsa-miR-6876-5p	hsa-miR-3202<br>hsa-miR-185-5p<br>hsa-miR-6891-5p<br>hsa-miR-12118<br>hsa-miR-4644<br>hsa-miR-6876-5p<br>hsa-miR-5192<br>hsa-miR-4533<br>hsa-miR-4476<br>hsa-miR-4306<br>hsa-miR-3173-3p<br>hsa-miR-6888-5p<br>hsa-miR-4428<br>**hsa-miR-4311**<br>hsa-miR-4496<br>hsa-miR-12124<br>hsa-miR-6856-5p<br>hsa-miR-6758-5p<br>hsa-miR-583<br>hsa-miR-1276	**hsa-miR-4311**<br>hsa-miR-1252<br>hsa-miR-5192<br>hsa-miR-5093<br>hsa-miR-4428<br>has-miR-3676-5p
rs149238335	170022800	hsa-miR-3121-5p	hsa-miR-1914-5p<br>hsa-miR-3074-5p<br>hsa-miR-7152-5p	hsa-miR-501-5p<br>hsa-miR-543<br>hsa-miR-4501<br>hsa-miR-5008-3p<br>hsa-miR-3074-5p<br>hsa-miR-30e-5p<br>hsa-miR-3164
rs192326883	170023009	hsa-miR-5571-3p<br>hsa-miR-6800-5p	hsa-miR-3682-3p<br>hsa-miR-6800-5p<br>hsa-miR-6802-5p	hsa-miR-5571-3p<br>hsa-miR-331-5p<br>hsa-miR-384<br>hsa-miR-4777-5p

				hsa-miR-1245b-3p<br>hsa-miR-4770<br>hsa-miR-4305<br>hsa-miR-143-3
rs3914146	170023020	hsa-miR-497-3p<br>hsa-miR-495-3p<br>hsa-miR-5688	hsa-miR-5688<br>hsa-miR-7-1-3p<br>hsa-miR-7-2-3p<br>hsa-miR-495-3p<br>hsa-miR-497-3p	hsa-miR-6715a-3p<br>hsa-miR-5688<br>hsa-miR-7-1-3p<br>hsa-miR-4658<br>hsa-miR-4704-5p
rs3914145	170023041	hsa-miR-4284<br>hsa-miR-4417<br>**hsa-miR-4437**<br>hsa-miR-4674<br>hsa-miR-296-5p<br>**hsa-miR-4301**<br>hsa-miR-6724-5p<br>hsa-miR-6773-5p	**hsa-miR-4301**<br>hsa-miR-3136-3p<br>hsa-miR-6773-5p<br>hsa-miR-296-5p<br>hsa-miR-7155-3p<br>hsa-miR-6724-5p<br>hsa-miR-3620-5p<br>hsa-miR-4284<br>hsa-miR-10400-3p<br>hsa-miR-1304-3p<br>**hsa-miR-4437**<br>hsa-miR-1587<br>hsa-miR-4674<br>hsa-miR-24-3p	hsa-miR-4296<br>hsa-miR-4265<br>hsa-miR-4322<br>hsa-miR-4284<br>hsa-miR-4417<br>**hsa-miR-4437**<br>**hsa-miR-4301**<br>hsa-miR-4283<br>hsa-miR-1178-3p<br>hsa-miR-4674<br>hsa-miR-378g
rs147435942	170023051	hsa-miR-18a-5p<br>hsa-miR-18b-5p<br>**hsa-miR-4521**<br>hsa-miR-4735-3p<br>hsa-miR-548n	hsa-miR-548m<br>hsa-miR-12135<br>hsa-miR-1264<br>hsa-miR-3184-3p<br>**hsa-miR-4521**	hsa-miR-513-5p<br>hsa-miR-18b-5p<br>hsa-miR-4735-3p<br>hsa-miR-3974<br>hsa-miR-4468<br>hsa-miR-527<br>**hsa-miR-4521**<br>hsa-miR-518a-5p<br>hsa-miR-4422
rs189382403	170023169	hsa-miR-6798-3p	hsa-miR-126-3p<br>hsa-miR-4520-5p	hsa-miR-1208<br>hsa-miR-4720-3p<br>hsa-miR-4804-3p<br>hsa-miR-93-3<br>hsa-miR-4420
rs9824427	170023396	**hsa-miR-5700**	hsa-miR-664b-3p	**hsa-miR-5700**

		**hsa-miR-586**<br>**hsa-miR-369-3p**<br>hsa-miR-374a-5p<br>hsa-miR-374b-5p<br>hsa-miR-374c-5p<br>hsa-miR-5692b<br>hsa-miR-5692c<br>hsa-miR-655-3p<br>hsa-miR-181a-5p<br>hsa-miR-181b-5p<br>hsa-miR-181c-5p<br>hsa-miR-181d-5p<br>hsa-miR-3143<br>hsa-miR-4262<br>hsa-miR-543<br>hsa-miR-595	hsa-miR-579-3p<br>hsa-miR-5692c<br>**hsa-miR-369-3p**<br>hsa-miR-4760-3p<br>**hsa-miR-5700**<br>**hsa-miR-586**<br>hsa-miR-33a-5p<br>hsa-miR-155-5p<br>hsa-miR-33b-5p	**hsa-miR-369-3p**<br>hsa-miR-374b-5p<br>hsa-miR-374c-5p<br>hsa-miR-543<br>hsa-miR-4666a-5p<br>hsa-miR-340-5p<br>hsa-miR-374a-5p<br>**hsa-miR-586**<br>hsa-miR-502-3p

**Table S7.** detail of transcription binding factors in wild type and mutant type of SNPs in 5′ UTR region.			
No.	Transcription binding Factors	No.	Transcription binding Factors
1. rs994884642			
0	GR-alpha	0	GR-alpha
1	Ap-2alpha	1	Ap-2alpha
2	GCF	2	GCF
3	E2F-1	3	E2F-1
4	STAT4	4	STAT4
5	c-ETs	5	c-ETs
6	Egr-3	6	Pax-5
7	Pax-5	7	P53
8	P53	8	WT1
9	WT1		
2. rs1482898617			
0	GR-alpha	0	GR-alpha
1	RXR-alpha	1	E2F-1
2	E2F-1	2	GCF
3	GCF	3	STAT4
4	STAT4	4	c-ETs-1
5	c-ETs-1	5	Egr-3
6	Egr-3	6	Pax-5
7	Pax-5	7	P53
8	P53	8	AP-2alphaA
9	AP-2alphaA		
3. rs1024270582			
0	GR-alpha	0	GR-alpha
1	AP-2alphaA	1	AP-2alphaA
2	GCF	2	E2F-1
3	E2F-1	3	Egr-3
4	Egr-3	4	Pax-5
5	Pax-5	5	p53
6	p53	6	Sp1
7	WT1		
8	SP1		
4. rs1031689697			
0	GR-alpha	0	GR-alpha
1	AP-2alphaA	1	AP-2alphaA
2	GCF	2	E2F-1
3	E2F-1	3	Egr-3
4	Egr-3	4	Pax-5
5	Pax-5	5	p53
6	p53	6	Sp1
7	WT1		
8	SP1		

5. rs1366285245			
0	GCF	0	GCF
1	Pax-5	1	Pax-5
2	P53	2	P53
3	WT1	3	TFII-I
4	E2F-1	4	E2F-1
5	ETF	5	RXR-alpha
6	RXR-alpha	6	C/EBPbeta
7	C/EBPbeta	7	GR-alpha
8	GR-alpha	8	AP-2alpha
9	AP-2alpha		
6. rs111868479			
0	GCF	0	GCF
1	Pax-5	1	Pax-5
2	P53	2	P53
3	WT1	3	WT1
4	E2F-1	4	RXR-alpha
5	ETF	5	C/EBPbeta
6	RXR-alpha	6	FOXP3
7	C/EBPbeta	7	GR-alpha
8	FOXP3	8	AP-2alpha
9	GR-alpha		
10	AP-2alphaA		
7. rs763083643			
0	GCF	0	GCF
1	Pax-5	1	Pax-5
2	P53	2	P53
3	WT1	3	RXR-alpha
4	E2F-1	4	C/EBPbeta
5	ETF	5	GR-alpha
6	RXR-alpha	6	FOXP3
7	C/EBPbeta	7	AP-2alpha
8	GR-alpha		
9	FOXP3		
10	AP-2alphaA		
8. rs766667625			
0	GCF	0	GCF
1	Pax-5	1	Pax-5
2	P53	2	P53
3	WT1	3	WT1
4	E2F-1	4	RXR-alpha
5	ETF	5	C/EBPbeta
6	RXR-alpha	6	GR-alpha
7	C/EBPbeta	7	AP-2alpha
8	GR-alpha	8	FOXP3
9	AP-2alphaA		
10	FOXP3		
9. rs778557075			
0	C/EBPbeta	0	C/EBPbeta
1	GR-alpha	1	GR-alpha
2	AP-2alphaA	2	XBP-1

3	XBP-1	3	RXR-alpha
4	RXR-alpha	4	TFII-I
5	TFII-I	5	MAZ
6	MAZ	6	FOXP3
7	WT1	7	AP-2alphaA
8	E2F-1		
9	FOXP3		
10. rs968409340			
0	C/EBPbeta	0	C/EBPbeta
1	GR-alpha	1	GR-alpha
2	AP-2alphaA	2	AP-2alphaA
3	XBP-1	3	XBP-1
4	RXR-alpha	4	RXR-alpha
5	TFII-I	5	TFII-I
6	MAZ	6	FOXP3
7	WT1		
8	E2F-1		
9	FOXP3		
11. rs750297755			
0	C/EBPbeta	0	C/EBPbeta
1	GR-alpha	1	GR-alpha
2	AP-2alphaA	2	AP-2alphaA
3	XBP-1	3	XBP-1
4	RXR-alpha	4	RXR-alpha
5	PPAR-alpha: RXR-alpha	5	PPAR-alpha: RXR-alpha
6	TFII-I	6	TFII-I
7	MAZ	7	E2F-1
8	WT1	8	FOXP3
9	E2F-1		
10	FOXP3		
12. rs542458816			
0	C/EBPbeta	0	C/EBPbeta
1	GR-alpha	1	GR-alpha
2	AP-2alphaA	2	AP-2alphaA
3	XBP-1	3	XBP-1
4	RXR-alpha	4	RXR-alpha
5	PPAR-alpha: RXR-alpha	5	PPAR-alpha: RXR-alpha
6	TFII-I	6	TFII-I
7	MAZ	7	Pax-5
8	WT1	8	P53
9	E2F-1	9	AhR
10	FOXP3	10	AhR-Amt
		11	WT1
		12	E2F-1
		13	ETF
		14	FOXP3
